# Portable Sleep Apnea Syndrome Screening and Event Detection Using Long Short-Term Memory Recurrent Neural Network

**DOI:** 10.3390/s20216067

**Published:** 2020-10-25

**Authors:** Hung-Chi Chang, Hau-Tieng Wu, Po-Chiun Huang, Hsi-Pin Ma, Yu-Lun Lo, Yuan-Hao Huang

**Affiliations:** 1 Department of Electrical Engineering, National Tsing Hua University, Hsinchu 30013, Taiwan; Scott_Chang@asus.com (H.-C.C.); pchuang@ee.nthu.edu.tw (P.-C.H.); hp@ee.nthu.edu.tw (H.-P.M.); 2 Department of Mathematics and Department of Statistical Science, Duke University, Durham, NC 27708, USA; hauwu@math.duke.edu; 3 Department of Thoracic Medicine, Healthcare Center, Chang Gung Memorial Hospital, School of Medicine, Chang Gung University, Taipei 33302, Taiwan

**Keywords:** abdominal movement signal, hypopnea, LSTM-RNN, neural network, oxygen saturation, sleep apnea syndrome, sleep–wake detection, synchrosqueezing transform, triaxial accelerometer, thoracic movement signal

## Abstract

Obstructive sleep apnea/hypopnea syndrome (OSAHS) is characterized by repeated airflow partial reduction or complete cessation due to upper airway collapse during sleep. OSAHS can induce frequent awake and intermittent hypoxia that is associated with hypertension and cardiovascular events. Full-channel Polysomnography (PSG) is the gold standard for diagnosing OSAHS; however, this PSG evaluation process is unsuitable for home screening. To solve this problem, a measuring module integrating abdominal and thoracic triaxial accelerometers, a pulsed oximeter (SpO_2_) and an electrocardiogram sensor was devised in this study. Moreover, a long short-term memory recurrent neural network model is proposed to classify four types of sleep breathing patterns, namely obstructive sleep apnea (OSA), central sleep apnea (CSA), hypopnea (HYP) events and normal breathing (NOR). The proposed algorithm not only reports the apnea-hypopnea index (AHI) through the acquired overnight signals but also identifies the occurrences of OSA, CSA, HYP and NOR, which assists in OSAHS diagnosis. In the clinical experiment with 115 participants, the performances of the proposed system and algorithm were compared with those of traditional expert interpretation based on PSG signals. The accuracy of AHI severity group classification was 89.3%, and the AHI difference for PSG expert interpretation was 5.0±4.5. The overall accuracy of detecting abnormal OSA, CSA and HYP events was 92.3%.

## 1. Introduction

According to a recent report [1], 13% men and 6% women between the ages of 30 and 70 years are affected by obstructive sleep apnea-hypopnea syndrome (OSAHS). Patients suffering from OSAHS have symptoms such as excessive daytime sleepiness, morning headache, hypertension and decreased libido [2]. However, people are often unaware of OSAHS because apnea/hypopnea events only occur during sleep. According to the American Academy of Sleep Medicine scoring manual [3], an apnea event is identified when a drop of 90% respiratory airflow lasts for at least 10 s. Moreover, a hypopnea event is defined when a drop of over 30% respiratory airflow lasts for at least 10 s with at least 3% associated decrease in oxygen saturation (SpO_2_) or arousal from sleep. The apnea-hypopnea index (AHI), which is defined as the total number of apnea and hypopnea events per hour of sleep, is a vital metric to quantize the severity of sleep breathing disorder. Although AHI is recently criticized and other phenotype information of sleep breathing problems should be considered in clinical diagnosis [4], it still is a reliable metric for SDB screening at home before the patients are recommended for other decent testing or diagnosis in hospital. Full-channel polysomnography (PSG) is the traditional method of diagnosing OSAHS. In PSG, various physical and biological signals containing sleep information are comprehensively recorded. Although PSG is the standard for diagnosing OSAHS, it has several drawbacks. Subjects are required to wear numerous sensors (more than 20 channels) for monitoring the condition of the body during sleep. The PSG examination can be performed only in the hospital and the sleep quality of the patients can be influenced by several external constraints. Moreover, to diagnose an OSAHS patient, it usually requires more than 6 h for a doctor or sleep technician to observe multichannel and overnight PSG signals and to label the sleep breathing events accordingly. Therefore, PSG measurement and diagnosis are expensive, time consuming and unsuitable for large-scale home-based screening. Several solutions have been proposed to alleviate this difficulty. A common solution is reducing the number of sensors. Several issues on the ambulatory monitoring for obstructive sleep apnea syndrome were raised by [5], and guidelines of using critical channels were also provided for sleep disorder diagnosis and management. In general, these solutions are classified into four classes [6]. A Level-III or Level-IV solution is considered in this paper.

In the past decade, several reduced-channel technologies have been developed to evaluate OSAHS severity. Multiple biological signals, such as electrocardiogram (ECG) [7], ballistocardiography [8], SpO_2_ [9], respiratory efforts [10] and snoring sounds [11], have been used to derive statistical or instantaneous signal features that are highly related with apnea events for sleep event identification. With the derived features from the selected sensors, various automatic annotation algorithms have been developed for sleep apnea events. Classification levels are of two types—the AHI level and event level. At the AHI level, the AHI of the whole night sleep is estimated for diagnosis. At the event level, each single apnea and hypopnea event is identified and classified and hence the AHI is accordingly calculated for the diagnosis. For the classification, various machine learning techniques such as support vector machine (SVM) [12], ensemble classifiers [13], and Bayesian network-based classifier [14] have been used to identify the sleep apnea events. Recently, a convolutional-neural-network-based deep learning framework [15] was proposed to detect obstructive sleep apnea events. In another study [7], the hidden-Markov-model-based deep neural network was used for detecting sleep apnea based on ECG signals. Raw biological signals without feature extraction have been used in several studies for detecting sleep apnea events through deep learning [9]. Quiceno-Manrique [16], Mendez [17], De Chazal [13] and Novak [18] used ECG signal for diagnosing OSAHS. In [10,19], abdominal (ABD) and thoracic (THO) movements were proven to be excellent parameters for diagnosing OSA occurrence. In [20], multiple channels, SpO_2_ and photoplethysmography (PPG) were used to estimate the blood volume changes for OSA prediction. For other literature works, refer to [21,22]. The primary differences and contributions of this study are presented as follows:

- This study is the first work to apply two triaxial accelerometers, a single-lead ECG and a finger oximeter for portable sleep apnea syndrome screening. The clinic experiments were performed by recording overnight signals of suspected patients under the approval of Institutional Review Board of hospital. Most works designed new sensing devices without clinic experiments or developed new detection algorithms by using public database.
- This work proposes a complete systematic framework of sensing devices and algorithms to detect and identify OSA, central sleep apnea (CSA) and hypopnea (HYP) events. This system can not only evaluate AHI values but also provide reliable event-level classification results of various sleep apnea events. Except our previous work [23] based on piezo-electronic bands, most works can only detect OSA events and evaluate AHI only.

In this study, a hardware solution was combined with a novel neural-network-based classification technique for identifying OSA, CSA and HYP events and NOR breathing by using two triaxial accelerometers (TAA), a pulsed oximeter and ECG. The proposed classification algorithm performs event level prediction but not the AHI level prediction. The features of the abdominal TAA (ABD-TAA), thoracic TAA (THO-TAA) and SpO_2_ signals were extracted from the recorded signals. Then, a modified long short-term memory recurrent neural network (LSTM-RNN) was proposed to classify the OSA, CSA and HYP events and NOR breathing in the overnight recorded signals. To avoid underestimation of the AHI from the predicted apnea and hypopnea events, the sleep–wake status was predicted by analyzing the ECG signal with a CNN classifier. The AHI severity group classification, AHI difference and OSA/CSA/HYP event and normal breathing classification were also analyzed to demonstrate the superiority of the proposed OSAHS screening system.

This study aimed to develop an unattended sleep apnea screening system that can be incorporated in the personal healthcare services with less labeling labor. The proposed screening system can be applied to evaluate the long-term sleep breathing performance of the potential subjects. These devices should be used in patients with a high pretest probability for obstructive sleep apnea/hypopnea syndrome according to 2007 AASM guideline (Reference 4) for the home-base diagnosis test. Patients suspected with respiratory, cardiologic and neurologic disorders should be excluded in this test. Primary care physician or sleep specialist would be the one who arranges this test.

## 2. Material and Methods

### 2.1. Material

The THO and ABD movements were recorded using piezo-electric bands at a sampling rate of 100 Hz on the Alice 5 PSG acquisition system (Philips Respironics, Murrysville, PA, USA). The SpO_2_ signals were also recorded at a sampling rate of 1 Hz in the PSG signals. The OSA, CSA and HYP events and NOR breathing were identified and labeled by sleep experts in the PSG signals as the reference classifier. At the same time as the PSG recoding process, the proposed THO-TAA, ABD-TAA and ECG sensing devices were also attached to the chest and abdomen of the participant for capturing the signals required for the proposed AHI evaluation system. Polysomnography (Alice 5, Respironics) was performed on all patients using standard techniques. Sleep stages and arousals were scored according to the AASM criteria [3]. Respiratory efforts were measured by piezo-electric bands, and arterial oxygen saturation was measured by pulse oximetry.

Established criteria were used to score respiratory events such as hypopnea, obstructive apnea, central apnea and mixed type apnea [3] during sleeping time. Apnea was defined as nasal flow cessation for more than 10 s. It was scored as obstructive (OSA) if the paradoxical respiratory and abdominal efforts were observed. It was scored as central (CSA) if none of these excursions were observed. It was scored as mixed if this effort is resumed toward the end of the period of apnea. The mixed type apnea was classified as OSA in this work because of its similar contribution factors to OSA. Hypopnea (HYP) was defined as a 30% reduction in nasal pressure transducer followed by an arousal or more than 3% decrease in SpO_2_. In this work, a segment signal was scored as normal if none of the above-mentioned events was identified.

### 2.2. Integrated Sensing System

**Figure 1:**
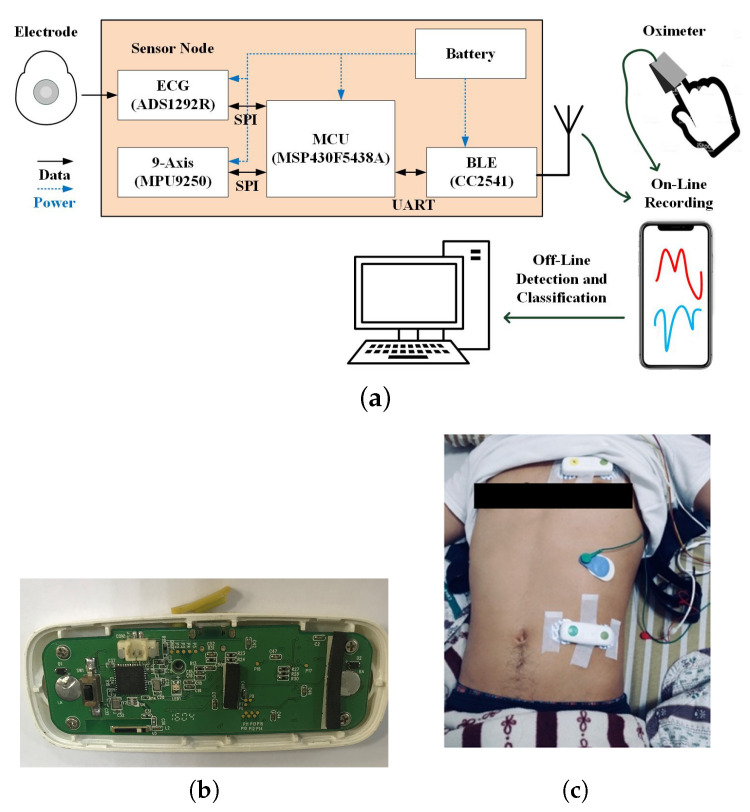
(**a**) Block diagram of the integrated sensing device and system; (**b**) photo of the sensing device; and (**c**) devices worn for sensing the ABD-TAA, THO-TAA and ECG signals.

Figure 1a depicts the proposed integrated sensing system that captures biomedical signals for sleep event detection/classification and AHI evaluation. A 27-g sensing device was devised and fabricated with a nine-axis accelerometer, an ECG sensor, a Bluetooth module and a microcontroller (Figure 1b). The sensing device included an ultra-low-power microcontroller (MSP430) that controlled MPU9250 to capture TAA signals, which were then delivered to a mobile device, such as smartphone or tablet, through Bluetooth module CC2541. The integrated sensing system could continuously sense and record signals for 34 h with a 300 mAh battery. The signal word-length and sampling rate are 12 bits/500 Hz and 16 bits/50 Hz for the ECG and accelerator, respectively. The transmission baud rate from a sensor device to iOS device is 115,200 bps. The bandwidth and reliability was verified to be sufficient for continuous transmission of the overnight ECG and acceleration signals. The reconnection procedure was also implemented in the Bluetooth link in case that patients might wake up and leave the transmission coverage, for example to go to restroom at night.

In the clinical experiment, two integrated TAA/ECG sensing devices were attached on the chest and abdomen of the participants and the ECG electrode was attached to the chest (Figure 1c). To record respiratory information, one sensing device was placed from the left parasternal line, 4th or 5th intercostal space to the mid-clavicle line to measure the maximal thoracic movement. The other sensing device was placed from the left subcostal anterior axillary line to the umbilical area to measure the maximal abdomen movement. In this way, we not only obtained strong thoracic and abdomen movement signal but also strong EKG signal. In the proposed recording and storage system, a prototype app software with graphic user interface on iOS device was built to control the progress of the data recording. All the sensed physiological signals were transmitted from the sensing devices to the iOS device through Bluetooth. Then, they were uploaded to the Dropbox cloud data server for the following data analysis.

### 2.3. Signal Preprocessing

**Figure 2:**
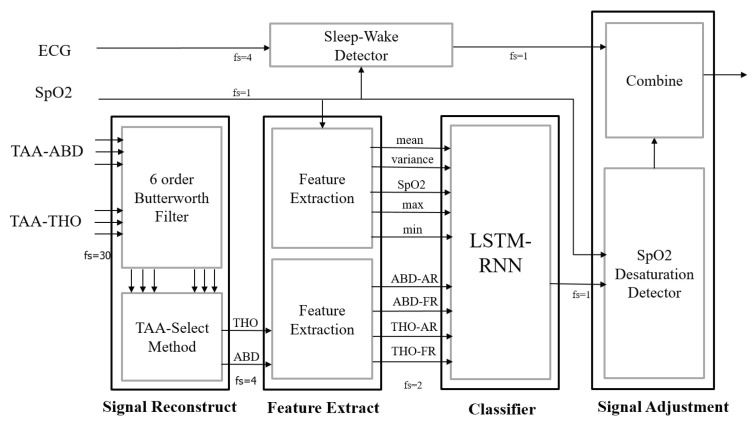
Processing diagram of the proposed AHI evaluation system.

Figure 2 displays the processing diagram of the proposed AHI evaluation system. Six channels of the ABD-TAA and THO-TAA signals were passed through six-order low-pass filters with a 0.8-Hz cut-off frequency and then converted into two respiratory motion signals, namely THO and ABD. Subsequently, the THO and ABD signals were segmented by a 10-s window and the SpO_2_ signal was segmented by a 20-s window. Nine features in each segment were generated. These features were used to classify four types of sleep breathing events with an LSTM-RNN classifier. SpO_2_ desaturation and sleep–wake detectors were used to improve the results of the LSTM-RNN classifier for the AHI evaluation. The algorithm is detailed step by step as follows.

**Figure 3:**
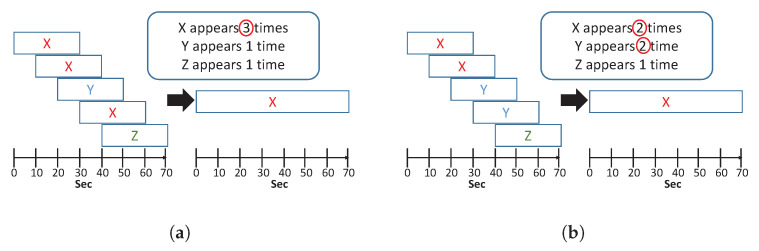
(**a**) TAA selection with the most appearances of one axis; and (**b**) TAA selection with equal appearances of two axes.

Each TAA sensor sampled a three-axis acceleration vector at a time. Typically, principle component analysis (PCA) is used to combine the three-dimensional (3D) acceleration vector into 1D signal for the following analysis. Although PCA is suitable when the recording time is short, this approach is insufficient for overnight recording. The PCA could possibly distort the useful information of sleep breathing features because of the nonstationarity, particularly when the selected axis is switched frequently because of change in the sleep position. Thus, a TAA selection method was proposed to avoid this problem as shown in Figure 3. Three-dimensional TAA signals were first segmented by 30-s window with a 10-s time step. The number of periodic peaks was counted in a segment of an axis. Then, the axis with the most similar number of peaks to the human average respiration rate (6–9 peaks per 30 s) was selected as the output axis. After determining the selected axes of five successive segments, the most frequent axis in the previous five segments was selected as the output signal for the following analysis, as depicted in Figure 3a. If two axes had equal appearances, the axis with the larger magnitude was selected, as depicted in Figure 3b.

### 2.4. Feature Extraction

The preprocessed 1D THO and ABD signals were used to generate the features for sleep breathing event classification. The most obvious feature of the OSA event is the paradox between the THO and ABD signals. For the CSA event, the signal strengths of the THO and ABD signals are extremely small and exhibit small frequency deviation (e.g., the cardiogenic artifact). Because distinguishing HYP events in the ABD and THO signals is difficult, SpO_2_ is incorporated to detect the HYP events.

#### 2.4.1. Features of the THO and ABD signals

The THO and ABD signals are denoted as $Y_{tho}$ and $Y_{abd}$, respectively. The THO and ABD signals were segmented using a 10-s window with a step of 0.5 s for feature extraction. According to the aforementioned physiological properties of the OSA, CSA and HYP events, the amplitude ratios (ARs) and the frequency ratios (FRs) [23] were considered as follows:

(1) $$\begin{array}{c} AR_{tho}\left(n\right)=\frac{Q_{95}\left({\tilde{A}}_{tho}\left(t\right)\chi_{CW(n)}\right)}{Q_{95}\left({\tilde{A}}_{tho}\left(t\right)\chi_{PW(n)}\right)}\\ AR_{abd}\left(n\right)=\frac{Q_{95}\left({\tilde{A}}_{abd}\left(t\right)\chi_{CW(n)}\right)}{Q_{95}\left({\tilde{A}}_{abd}\left(t\right)\chi_{PW(n)}\right)}. \end{array}$$

(2) $$\begin{array}{c} FR_{tho}\left(n\right)=\log_{10}\left(\frac{\int_{0.8}^{1.5}{\left|F(Y_{tho}\left(t\right)\chi_{CW(n)})\left(\xi \right)\right|}^{2}d\xi }{\int_{0.1}^{0.8}{\left|F(Y_{tho}\left(t\right)\chi_{CW(n)})\left(\xi \right)\right|}^{2}d\xi }\right)\\ FR_{abd}\left(n\right)=\log_{10}\left(\frac{\int_{0.8}^{1.5}{\left|F(Y_{abd}\left(t\right)\chi_{CW(n)})\left(\xi \right)\right|}^{2}d\xi }{\int_{0.1}^{0.8}{\left|F(Y_{abd}\left(t\right)\chi_{CW(n)})\left(\xi \right)\right|}^{2}d\xi }\right) \end{array}$$

where χ is the indicator function (1 or 0) for the windowing segmentation of input signals; $Q_{95}$ represents the 95% quantile of the given function; ${\tilde{A}}_{tho}\left(t\right)$ and ${\tilde{A}}_{abd}\left(t\right)$ are the amplitudes of the THO and ABD signals, respectively, which were determined using the synchrosqueezing transform; and F represents the Fourier transform. CW represents the current window, that is, the *n*th CW is denoted as CW(n)⊂R, where *n* is the index of segment. PW is the previous 60-s windowed signal before the current window. PW contains the baseline amplitude for AR. The *n*th PW associated with the *n*th CW is denoted as PW(n)⊂R. Consequently, $AR_{tho}\left(n\right)$ and $AR_{abd}\left(n\right)$ represent the ARs and $FR_{tho}\left(n\right)$ and $FR_{abd}\left(n\right)$ represent the FRs of the THO and ABD signals, respectively, over the *n*th CW.

Synchrosqueezing transform (SST) is a novel nonlinear-type time–frequency analysis technique aiming to analyze complicated and nonstationary time series. It has been theoretically proved to enjoy several nice properties [24,25]. For our application, the main benefit of SST is an accurate estimation of the instantaneous frequency and the amplitude modulation of the respiratory signal. Moreover, the estimation does not depend on whether or not the oscillatory patter or wave-shape function is sinusoidal [26]. In addition, the SST is robust to various kinds of noise, including colored or even nonstationary random process [25].

The AR features were determined from the estimated amplitudes of the THO and ABD signals, which are denoted as ${\tilde{A}}_{tho}\left(t\right)$ and ${\tilde{A}}_{abd}\left(t\right)$, respectively, by using the synchrosqueezing transform. This step is critical because it suppresses the artifacts caused by the sudden change of body posture. The FR indicates the frequency distributions of the respiration and probably the cardiogenic artifact caused by heart beats. The integration range from 0.8 to 1.5 Hz in the numerator in (2) is the average range of heart beat rate. In our algorithm, the heart beat information was taken into account and the cardiogenic artifact indicates how silent the respiratory signal is. The detailed properties of the ARs and FRs of the THO and ABD signals can be obtained from [23].

#### 2.4.2. Features of SpO${}_{2}$ Signal

**Figure 4:**
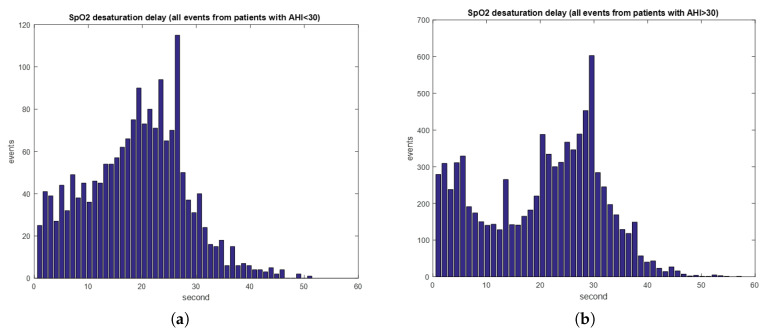
(**a**) SpO_2_ desaturation time of patients with AHI lower than 30; and (**b**) SpO_2_ delay time of patients with AHI higher than 30.

**Figure 5:**
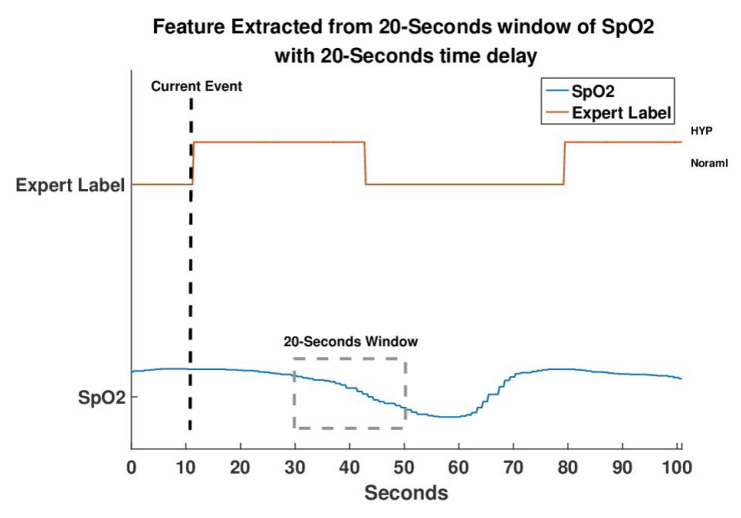
The decline of the SpO_2_ signal occurs 20–40 s after abnormal events according to the physiological phenomenon.

SpO_2_ is the percentage of oxyhemoglobin in hemoglobin. When sleep apnea and hypopnea events occur, SpO_2_ decreases gradually until the subject breathes again. According to our data, the average delay time between an apnea (hypopnea) event and the 3% drop of SpO_2_ was 19.3±9.6 s. The average event duration is 20.2±3.4. Figure 4a,b displays the distributions of the desaturation delay times of all events for the patients with AHI > 30 and AHI < 30, respectively. For patients with severe symptoms (AHI > 30), the desaturation distribution exhibits a high probability of error in which the previous respiratory event related to desaturation is labeled as the current event, that is, the desaturation drop of the previous respiratory event is almost adjacent to the current event. Therefore, features of SpO_2_ were generated for every 20-s segment with a 20-s delay from the sampling point, as depicted in Figure 5. The minimum, maximum, mean and variance of the first derivative were used as the four features, and the original SpO_2_ signal was also reserved as the baseline. To eliminate the variation of subjects, the SpO_2_ signal was normalized by subtracting it by its median and dividing the obtained value by its standard deviation.

### 2.5. Neutral Network Model, Event Classification and AHI Evaluation

#### 2.5.1. Neural Network Model Classifier

**Figure 6:**
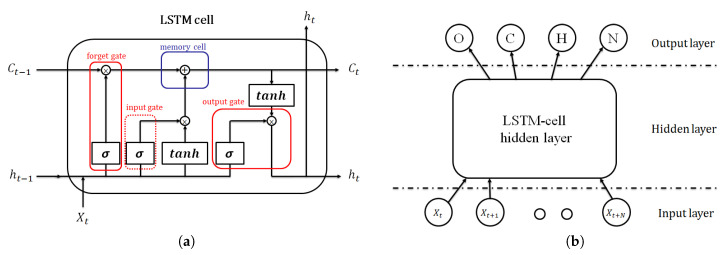
(**a**) $X_{t}$ is the *t*th input feature, where t=1,2,…,N. *N* is the total number of data points; $C_{t}$ is the *t*th memory; $h_{t}$ is the *t*th output; and σ and tanh represent the sigmoid and hyperbolic tangent function, respectively [27]. (**b**) LSTM-RNN architecture with the input layer, hidden layer and four-neuron output layer for classifying CSA, OSA, HYP and NOR states in every N seconds.

The RNN based on the LSTM model, which was first presented by Hochreiter [27], was instrumental in solving many sequence problems with long-term dependency, such as language translation, speech recognition, image captioning and genomic information learning [28,29,30,31]. The features of the sleep breathing events based on THO/ABD and SpO_2_ signals are time-varying and have long-term dependency. Therefore, an LSTM-RNN model was used to classify the sleep breathing events. The LSTM-RNN is an extension of the RNN and has more complex memory neurons than the RNN (Figure 6a). Unlike the original neuron with a simple loop in the RNN, every neuron in the RNN is replaced with an LSTM cell. An LSTM cell has three gates, namely the input, output and forget gates. These gates are scalars that are trained in every iteration to control the input, output and memory of every cell. Furthermore, the computation of output is reserved in the LSTM and combined with the new input. With the aforementioned design, the LSTM can thus deal with the long-term dependency problem, various desaturation times and many other subject variations for sleep breathing event classification.

**Figure 7:**
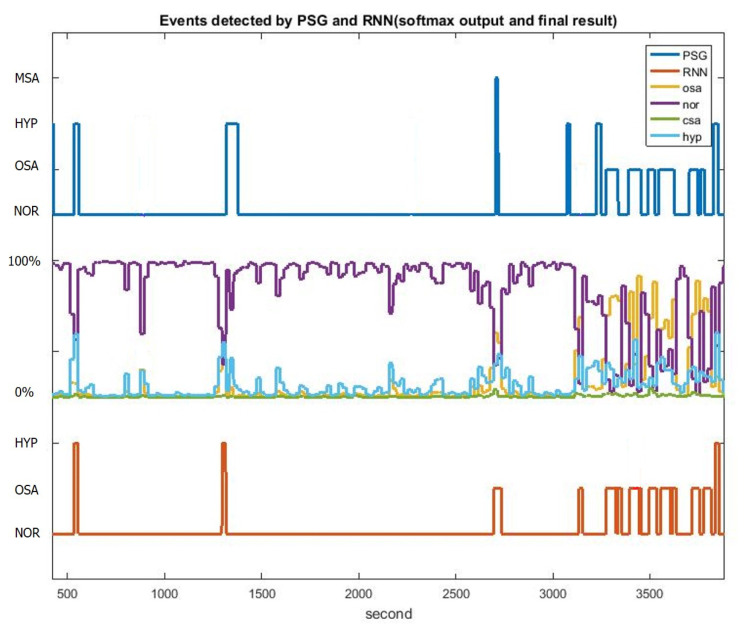
Event classification results of the PSG labeling, LSTM-RNN softmax outputs and final results of the LSTM-RNN classifier during 1-h sleep of a patient.

Figure 6b illustrates the LSTM-RNN architecture, which has three layers, namely the input, LSTM-cell hidden and output layer. The input layer consists of nine neurons corresponding to nine extracted features from the THO/ABD and SpO_2_ signals. The output layer contains four neurons representing four types of events, namely the OSA, CSA and HYP events and NOR breathing. The output of the network was normalized by using the softmax function. In total, 80 LSTM cells were utilized in the hidden layer according to the thumb of rule. The upper bound of the hidden neuron number was calculated by dividing the number of cases in the training dataset by the sum of the numbers of input and output layers in the network. The LSTM-RNN model was trained with 500 epochs of 500 batches of Adam gradient descents and a learning rate of 0.001. The activation function used in each layers was the rectified linear unit (ReLU) because of the benefit of sparsity and its capability of reducing the vanishing gradient. The loss function was used to compute the sum of cross entropy and L2 regularization with β=0.05. Moreover, gradient clipping was added to the loss function to avoid the exploding gradient. Figure 7 illustrates the event detection results of 1-h segment for a patient. The PSG labeling results obtained from experts are displayed in the top panel of Figure 7. The middle panel displays the softmax output results of the LSTM-RNN classifier. In this panel, the four curves represent the probabilities of the four types of events. The decision rule of the LSTM-RNN classifier involves selecting the event with the highest probability in every time step, as depicted in the red line in the bottom panel. The LSTM-RNN classifier generates almost the same event states as PSG labeling does.

#### 2.5.2. Oxygen Desaturation Detection

**Figure 8:**
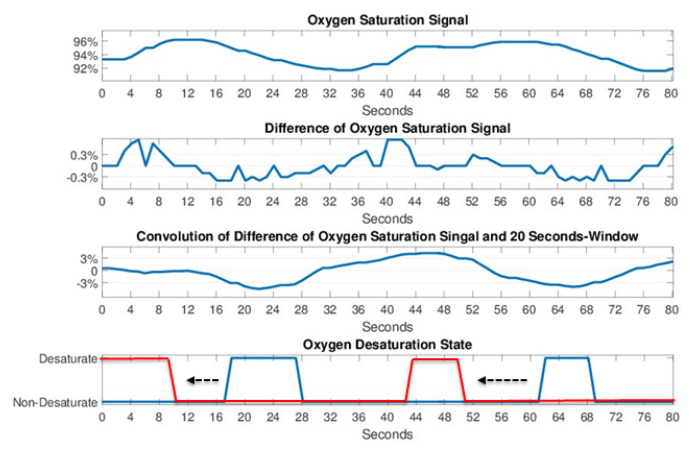
Processing steps for the oxygen desaturation detection.

**Figure 9:**
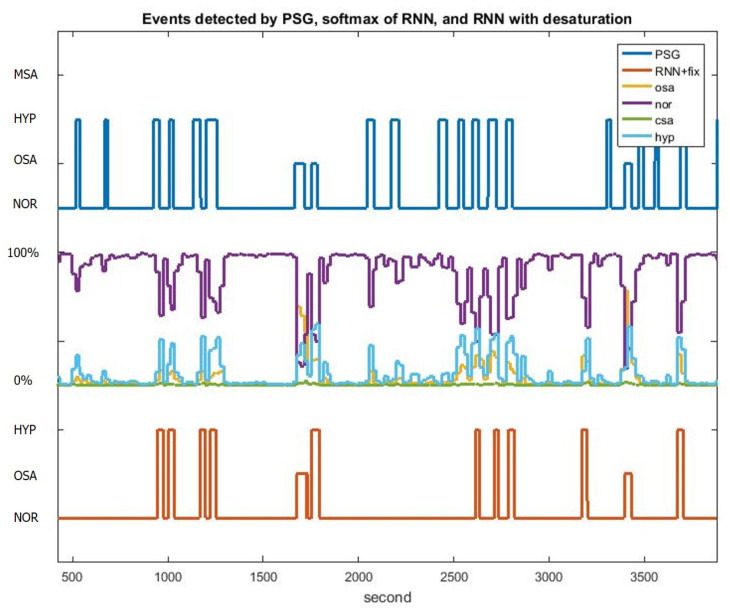
Event classification results of PSG labeling, the LSTM-RNN softmax outputs and the final results of the LSTM-RNN classifier with desaturation detection during 1-h sleep of a subject.

According to the 2014 guidelines from the American Academy of Sleep Medicine [3], a 3% drop of SpO_2_ is considered as a potential sleep apnea and hypopnea event. Therefore, the proposed sleep breathing event classifier incorporates a SpO_2_ desaturation detection scheme to capture every 3% drop in the SpO_2_ signal (Figure 8). First, the difference of SpO_2_ saturation signal was calculated and then convolved with a 20-s unity window to accumulate the difference. Afterwards, every desaturation with over 3% drop can be marked as an HYP event, which may not easily be detected using the LSTM-RNN classifier because limited CSA or OSA features can be extracted for the hypopnea event. Finally, the remarked signal was moved 20-s forward to compensate the delay of SpO_2_ desaturation. Figure 9 illustrates the 1-h classification results of PSG labeling, the softmax outputs of the RNN and the outputs of the LSTM-RNN classifier with desaturation detection. By adding SpO_2_ desaturation, the HYP softmax output exhibits higher probability than the NOR state. Therefore, the HYP events can be easily (see HYP softmax output) detected.

#### 2.5.3. Sleep–Wake Classification

Because AHI is defined as the number of apnea and hypopnea events that occur during sleep, heart rate variability (HRV) was used in this study to detect the sleep and wake status during overnight sleep [32]. According to a previous study [33], a CNN was used to classify the sleep and wake status by using the instantaneous heart rate (IHR) signal converted from the ECG signal and SpO_2_. Finally, the LSTM-RNN classification results and the sleep–wake state are combined to remove false positive events during the wake state. HRV is quantified by the intervals between successive heartbeats of ECG signals. HRV is estimated as the IHR per minute as follows:

(3) $$\mathrm{IHR}\left(r_{i}\right)=\frac{60}{r_{i}-r_{i-1}}\;i=2,\ldots ,n,$$

where $r_{i}$ denotes time instants in seconds when the *i*th R peak is detected. The unit of IHR is then beats per minute (bpm). Subsequently, the IHR signal along with the 20-s-delayed SpO_2_ signal was segmented into 30-s epochs for the CNN network.

**Figure 10:**
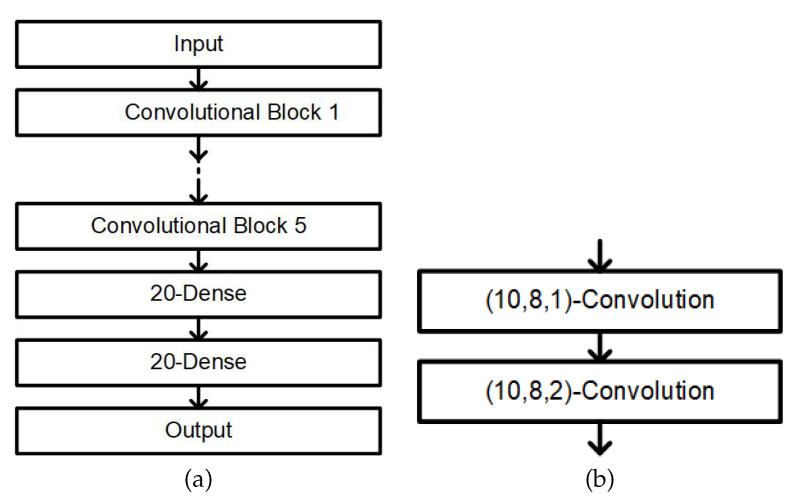
(**a**) Architecture of the one-dimensional CNN. The notation 20-Dense denotes that the fully connected layer possesses 20 nodes. For 5-min input signals, we used five convolution blocks [33]. (**b**) Architecture of a single convolution block. The notation (f,k,s)-convolution denotes that the convolutional layer has *f* filters with a kernel size *k* and stride *s*. The output of the block is half the size of the input [33]. A bias is added to the output of each filter, and the result is fed into a rectified linear unit (ReLU) activation function. A dropout with probability 0.5 is applied to the last layer and both fully-connected layers. The output of the network is normalized by the softmax function. An epoch is predicted to be wake if the output of the wake node is greater than or equal to that of the sleep node. We refer readers to Section 2.3 of [33] for more details.

Figure 10a displays the CNN network used to classify the sleep and wake state. The input is first passed through five convolution layers and then two fully connected layers. Figure 10b illustrates each convolution layer. A single convolution layer has ten filters with a kernel size 8, and the stride is equal to 1 and 2. Each fully connected layer has 20 nodes, and every node is associated with a bias and ReLU activation function. Finally, a softmax function is applied before the output layer. Five minutes of the IHR and SpO_2_ signals were used as inputs, which were normalized by subtracting the median value. The output was a 2D one-hot code for the sleep and wake states. L2 regularization was applied with β=0.3. The CNN network was trained using the Adam gradient descent with a learning rate of $10^{-3}$, a batch size of 100 and cross entropy as the loss function.

## 3. Results

The clinic experiments were approved by the Institutional Review Board of the Chang Gung Memorial Hospital (CGMH: No. 201601576B0). Clinical patients at the sleep center in CGMH, Linkou, Taoyuan, Taiwan who were suspected of having sleep apnea were considered for this study. In total, 115 participants were examined in the clinical experiments. The demographic details of the participants are summarized in Table 1. The sleep experts identified the OSA, CSA, mixed sleep apnea (MSA) and HYP events from the overnight PSG signals of all patients. The remaining signals were NOR states. The MSA was regarded as the OSA in this study because of the similarity of physiological features. The training and testing databases had nearly the same distribution over various severity levels, as presented in Table 2.

In our previous studies [23,34], SVM was used and followed by a state machine for screening OSAHS. The SVM model is divided into three types. First, the original SVM uses 50% of participants for training and 50% of participants for testing. Second, in the phenotype-based SVM [34], K=15 nearest subjects of all data are selected according to gender, BMI and age with weights of 4, 2 and 1, respectively. Third, in the phenotype-based SVM with comorbidity information, the most similar 20 subjects are first selected and then the nearest 15 subjects are selected from these candidates using the K-nearest neighborhood method. For the LSTM-RNN model, the time step *N* was first evaluated for screening OSAHS. The detection performances of various *N*s are presented in Table 3. When *N* was 20, the largest F1 score was 0.72±0.22 and the AHI difference was 8.1±7.3. As the time step increased, the performance declined because the average duration of all events (apnea and hypopnea) was approximately 20 s (Figure Table 3).

### LSTM-RNN with Oxygen Desaturation and Sleep–Wake Detection

Using the sleep–wake information of the overnight sleep, the classified sleep breathing events occurring when subjects were awake were eliminated. Thus, highly accurate sleeping hours for AHI evaluation could also be obtained in the experiment. Table 4 lists the sensitivity, precision, $F_{1}$ score and AHI difference for all subjects at various severity levels. We observed that sensitivity, precision and $F_{1}$ increased with the severity. The primary reason for this result was that the database size of the sleep breathing events for the severe group was considerably larger than that for the normal, mild and moderate groups. Compared with the generic SVM ($F_{1}$ score of 65%±26%) in Table 5, the proposed LSTM-RNN with oxygen desaturation and sleep–wake detection had a higher $F_{1}$ score (71%±22%) with respect to the PSG labeling of the sleep experts. The average AHI difference of the proposed LSTM-RNN model was 5.0±4.5, which is smaller than that of the generic SVM model. Table 6 lists the confusion matrix of the classification of the proposed LSTM-RNN model with oxygen desaturation and sleep–wake detection for different severity levels. The severity classification achieved an accuracy of 89.3%.

Table 7 presents the confusion matrix of the classification of OSA, CSA and HYP events and NOR breathing. The overall event-by-event classification accuracy was 83.3%. The NOR breathing and OSA events could be well identified, whereas the identification of HYP events was difficult because of the lack of obvious information for HYP events. Some CSA events were classified as OSA events mainly because the OSA events had more than twice the CSA events in the database. However, the accuracy of distinguishing abnormal events was still 92.3%. This detection accuracy approximates the recommended intra-class correlation (95%) for the reliability of different scorers by [35]. This difference is very close to traditional subjective interpretation. Therefore, the proposed portable sensing system and OSAHS event identification algorithm can be reliable for the OSAHS screening in the home environment.

## 4. Discussion

From the clinical perspective, through the proposed LSTM-RNN classifier with the TAA, ECG and SpO_2_ signals, the respiratory events (apnea vs. hypopnea) and pattern (obstructive vs. central) can be effectively detected by the proposed system. Moreover, sleep–wake status be identified by using a CNN algorithm with instantaneous heart rate derived from ECG and SpO_2_ signals. According to the heat rate and rhythm information in the ECG signal, our sensing devices and algorithm fully meet the requirements of AASM “SCOPER” (sleep, cardiovascular, oximetry, position, effort and respiratory) criteria for the home-base OSAHS detection. Compared with other Level 3 home-base equipments for sleep event screening using fewer sensors and channels for reducing sleep interference, the proposed sensing system and classification algorithm can provide better sleep quality and higher accuracy without sacrificing any useful information. The proposed system and algorithm can also support an effective early diagnosis and early treatment possibility for a clinically vital disease with high prevalence and low diagnostic and treatment rates.

From the hardware perspective, as suggested by a preliminary result provided in [33], the sleep–wake classification can be conducted accurately through PPG. This indicates that the ECG signal can be replaced by the PPG signal. Because only the partial information SpO_2_ of the PPG signal was considered, one channel can be reduced in the next-generation sensing device.

From the algorithmic perspective, the following aspects should be considered to further improve the algorithm. The signal quality was considered in this study. The robustness properties of the feature extraction algorithm was simply focused on avoiding the impact of the inevitable noise and artifact. In addition to using the existing signal quality index (SQI) for the ECG or PPG signal, a suitable SQI should be developed for the ABD-TAA and THO-TAA signals. By incorporating these indices into the algorithm, the algorithm performance should be improved. This possibility will be explored in future study.

### Limitation

This discussion is not complete without mentioning its limitation. First, the data were collected in a hospital environment designed for type I sleep screening. Additional data should be collected at the home-base environment to further confirm the applicability of the proposed model and algorithm. Another limitation is the database size. According to the encouraging positive results provided by the phenotype-based SVM, we expect to achieve a superior result if the database size increases. Specifically, with a larger database, we can have more cases with similar phenotype to build up an accurate model for each new-arriving patient.

## 5. Conclusions

In this study, a series of classification and detection algorithms was developed for screening sleep apnea patients by using a pulse oximeter and a wireless sensing system with TAA and ECG sensors. The features were extracted from the THO/ABD and SpO_2_ signals and then used for training the LSTM-RNN classifier. The proposed system incorporates an SpO_2_ desaturation detector and an ECG-based sleep–wake detector to improve the overall classification performance of the LSTM-RNN classifier. The severity group classification based on the AHI evaluation results of the proposed algorithm achieved an accuracy of 89.3%, and the sleep breathing event classification achieved an accuracy of 92.3%. Thus, we believe that the proposed screening system and classification algorithms can establish a solid foundation for the clinical screening of OSAHS.

This study has some potential future works. The proposed LSTM-based neural network has been proven to be effective in identifying several sleep apnea event types in this work. Since the proposed portable sensing system was designed for a homecare screening system, the LSTM-based neural network can be customized for individual person by using distributed learning techniques, which can be achieved by adopting phenotype information such as gender, weight, age and other personal physical information so as to enhance the personalized and high-accuracy sleep disorder screening system. Moreover, the proposed sensing system and APP software on a smartphone can record overnight data. To realize home-based screening or monitoring, the off-line data analysis (detection and event classification algorithms) on the PC can be further replaced by cloud-based analysis. That is, the user can upload the data by smartphone to a cloud server, and the data are then analyzed on the cloud server. Accordingly, the results can be easily viewed by the remote users such as doctors or caregivers.

## Figures and Tables

**Table 1:** Demographic details of the 115 participants.

Severity	Gender	AHI	BMI	Age	* TST	** SE	*** REM	**** NREM
		(times/hr)	(kg/m${}^{2}$)	(year)	(min)	(%)	(%)	(%)
Normal	all (9) male (6) female (3)	1.8 ± 1.6	23.2 ± 3.3	30.0 ± 8.2	312.9 ± 37.2	84.4 ± 9.9	15.4 ± 5.7	84.6 ± 5.7
Mild	all (17) male (13) female (4)	9.4 ± 2.4	25.6 ± 3.7	49.0 ± 11.3	313.4 ± 28.0	84.3 ± 7.3	17.8 ± 5.8	82.2 ± 5.8
Moderate	all (28) male (21) female (7)	21.7 ± 4.1	25.4 ± 2.7	48.5 ± 12.9	311.0 ± 40.7	83.9 ± 11.1	14.9 ± 5.6	85.1 ± 5.6
Severe	all (61) male (50) female (11)	61.1 ± 24.0	30.2 ± 6.0	50.3 ± 12.3	291.0 ± 52.6	79.4 ± 13.8	11.0 ± 6.2	89.0 ± 6.2

* TST, Total Sleep Time; ** SE, Sleep Efficiency; *** REM, Rapid Eye Movement Percentage; *** NREM, None Rapid Eye Movement Percentage.

**Table 2:** Distribution of the training and testing participants.

Level of Severity	Training Subjects	Testing Subjects	Total Subjects
Normal	6	3	9
Mild	9	8	17
Moderate	14	14	28
Severe	30	31	61
All levels	59	56	115

**Table 3:** Sensitivity, precision, $F_{1}$ score and AHI difference of LSTM-RNN with different time steps (*N*).

Time Step (*N*)	Precision	Sensitivity	*F*_1_ Score	AHI Difference
10 s	0.59±0.25	0.88±0.16	0.68±0.24	9.7±7.2
15 s	0.67±0.23	0.74±0.22	0.68±0.21	8.8±6.6
20 s	0.74±0.22	0.73±0.22	0.72±0.22	8.1±7.3
25 s	0.72±0.22	0.75±0.21	0.71±0.21	8.7±6.3
30 s	0.71±0.21	0.77±0.2	0.71±0.2	8.6±6.5

Precision = # of True Positive/(# of True Positive + # of False Positive); Sensitivity = # of True Positive/(# of True Positive + # of False Negative); *F*_1_ Score = (2 × Precision · Sensitivity)/(Precision + Sensitivity).

**Table 4:** Sensitivities, precisions, $F_{1}$ scores and AHI differences of LSTM-RNN with oxygen desaturation and sleep–wake detection for different severity groups.

LSTM-RNN Model	Sensitivity	Precision	*F*_1_ Score	AHI Difference
Normal	0.62±0.44	0.16±0.15	0.23±0.2	4.9±4.4
Mild	0.62±0.16	0.48±0.14	0.54±0.13	2.4±1.7
Moderate	0.64±0.2	0.77±0.15	0.68±0.16	5.8±5.7
Severe	0.81±0.13	0.86±0.07	0.83±0.09	5.7±4.0
All levels	0.73±0.2	0.74±0.23	0.71±0.22	5.0±4.5

**Table 5:** Comparison of the LSTM-RNN and SVM models with oxygen desaturation.

Model + Oxygen Desaturation	Original SVM	Phenotype SVM [34]	Phenotype SVM+ Comoribidity [34]	LSTM-RNN
Precision	0.77±0.25	0.74±0.27	0.74±0.28	0.72±0.22
Sensitivity	0.63±0.27	0.64±0.27	0.65±0.27	0.81±0.2
F1 score	0.65±0.26	0.65±0.26	0.65±0.29	0.72±0.22
AHI difference	10.6±13.4	9.8±10.0	9.0±11.8	6.0±5.2

**Table 6:** Confusion matrix of the LSTM-RNN model with oxygen desaturation and sleep–wake detection.

LSTM-RNN with Desaturation and Awake Detection		Expert Label			
		Normal	Mild	Moderate	Severe
RNN Label	Normal	3	0	0	0
	Mild	0	8	3	0
	Moderate	0	0	11	3
	Severe	0	0	0	28
Accuracy		89.28%			

**Table 7:** Confusion matrix of the OSA, CSA and HYP events and NOR breathing for the LSTM-RNN model with oxygen desaturation and sleep–wake detection.

Four Events Types Classification in LSTM-RNN		Expert Label			
		Normal	OSA	CSA	HYP
RNN Label	Normal	37,788	232	470	1824
	OSA	1391	7485	2132	1213
	CSA	21	35	1855	32
	HYP	556	1703	149	2138
Accuracy		83.34%			
